# A genome-wide CRISPR screen identified host genes essential for intracellular *Brucella* survival

**DOI:** 10.1128/spectrum.03383-23

**Published:** 2024-02-20

**Authors:** Heling Xu, Jingjing Lu, Fang Huang, Qi Zhang, Shuang Liu, Zeliang Chen, Shanhu Li

**Affiliations:** 1 Key Laboratory of Livestock Infectious Diseases in Northeast China, Ministry of Education, College of Animal Science and Veterinary Medicine, Shenyang Agricultural University, Shenyang, Liaoning, China; 2 Department of Cell Engineering, Beijing Institute of Biotechnology, Beijing, China; Yangzhou University, Yangzhou, China

**Keywords:** *Brucella*, genome-wide CRISPR screen, host-pathogen interaction, intracellular bacteria

## Abstract

*Brucella* is a zoonotic intracellular bacterium that poses threats to human health and economic security. Intracellular infection is a hallmark of the agent *Brucella* and a primary cause of distress, through which the bacterium regulates the host intracellular environment to promote its own colonization and replication, evading host immunity and pharmaceutical killing. Current studies of *Brucella* intracellular processes are typically premised on bacterial phenotype such as intracellular bacterial survival, followed by biochemical or molecular biological approaches to reveal detailed mechanisms. While such processes can deepen the understanding of *Brucella*-host interaction, the insights into host alterations in infection would be easily restricted to known pathways. In the current study, we applied CRISPR Cas9 screen to identify host genes that are most affected by *Brucella* infection on cell viability at the genomic level. As a result of CRISPR screening, we firstly identified that knockout of the negatively selected genes *GOLGA6L6*, *DEFB103B*, *OR4F29*, and *ERCC6* attenuate the viability of both the host cells and intracellular *Brucella*, suggesting these genes to be potential therapeutic targets for *Brucella* control. In particular, knockout of *DEFB103B* diminished *Brucella* intracellular survival by altering host cell autophagy. Conversely, knockout of positive screening genes promoted intracellular proliferation of *Brucella*. In summary, we screened host genes at the genomic level throughout *Brucella* infection, identified host genes that are previously not recognized to be involved in *Brucella* infection, and provided targets for intracellular infection control.

**IMPORTANCE:**

*Brucella* is a Gram-negative bacterium that infects common mammals causing arthritis, myalgia, neuritis, orchitis, or miscarriage and is difficult to cure with antibiotics due to its intracellular parasitism. Therefore, unraveling the mechanism of *Brucella*-host interactions will help controlling *Brucella* infections. CRISPR-Cas9 is a gene editing technology that directs knockout of individual target genes by guided RNA, from which genome-wide gene-knockout cell libraries can be constructed. Upon infection with *Brucella*, the cell library would show differences in viability as a result of the knockout and specific genes could be revealed by genomic DNA sequencing. As a result, genes affecting cell viability during *Brucella* infection were identified. Further testing of gene function may reveal the mechanisms of *Brucella*-host interactions, thereby contributing to clinical therapy.

## INTRODUCTION

The Brucellosis has become one of the world’s most common zoonoses involving a variety of mammals, causing economic losses in livestock and severe illness and death in human (1, 2). The microbes of *Brucella* species are rather easy to decontaminate by bleach, ultraviolet, or antimicrobial agents *in vitro* yet tricky to clear out in host organism infection because of its facultative intracellular parasitism (3). Multiple cells can be violated by *Brucella*, including professional phagocytes such as macrophages and dendritic cells and non-professionals like epithelial (4). Upon being taken by the host cell, *Brucella* initiate a life cycle of surviving, replicating, and propagating (5–7), with the regulation of bacterial type IV secretion system (T4SS) and lipopolysaccharide (8). Accordingly, the regulation of virulence gene expression and physical adaption to the host environment of *Brucella* was further studied to reveal the mechanism of *Brucella* parasitism (8).

Unlike the active discovery at the bacterial level, the host character in *Brucella* infection studies is normally result oriented, which means to identify a key gene in the host by a known pathway, an observed phenotype, or an interacting molecule and consequently to investigate the molecular mechanism. For example, revealing the survival and kinetics of intracellular *Brucellae* is derived from the phagocytosis of macrophages, where the engulfed microorganism is present in a membrane-bound vesicle that in turn fused with lysosomes (9, 10), only evading terminal lysosomal degradation (5, 6, 11). Host markers of the *Brucella*-containing vacuole (BCV) upon phagocytosis are also determined along the endocytic pathway, in the following order: the small GTPase Rab5 and the early endosomal antigen EEA-1 (12–14), the late endosomal LAMP1, LAMP2, CD63, and the small GTPase Rab7 that controls fusion with late endocytic compartments and lysosomes (6, 12, 13, 15). The intracellular localization of *Brucella* was revealed by microscopic imaging whereby the host endoplasmic reticulum (ER) was found to provide binding sites for BCV and facilitated intracellular bacterial replication (12, 16, 17). Further investigation of this stage yielded diverse mechanisms that promote *Brucella* propagation in terms of key molecules, signaling pathways, and organelle functionalities. For example, the small GTPase Sar1 activity directly controlled BCV binding to endoplasmic reticulum exit sites (ERES) (18, 19), the IRE1α pathway and its downstream unfolded protein response (UPR) caused dramatic restructuring of the ER (20–22), and the ER-Golgi secretory trafficking was crucial for *Brucella* replication (23–25). These findings have enhanced our understanding of the process of *Brucella* infection, but comprehensive and intuitive data from high-throughput screening of host genes are still lacking.

Genome-wide genetic screens using genome editing systems such as clustered regularly interspaced short palindromic repeats (CRISPR)/Cas9 have emerged as advanced tools to systematically characterize key genes elicit a specific function or phenotype for a cell type and have been applied in multiple fields such as identifying tumor vulnerability or drug sensitivity (26, 27), assessing new therapy (28), and revealing immune function (29). In infectious diseases, CRISPR screening functioned in identifying host factors crucial in pathogen infection (30, 31) or propagation (32) to provide new host-directed therapeutics, but no *Brucella* related screen has been done. Since the screen depending on cell viability (33), we sought to identify host genes engaged in restraining *Brucella* infection to facilitating cell proliferation or eliminating infected cells to impair *Brucella* colonization.

## RESULTS

### A genome-wide CRISPR-Cas9 screen identified host genes associated to *Brucella* infection

**Fig 1:**
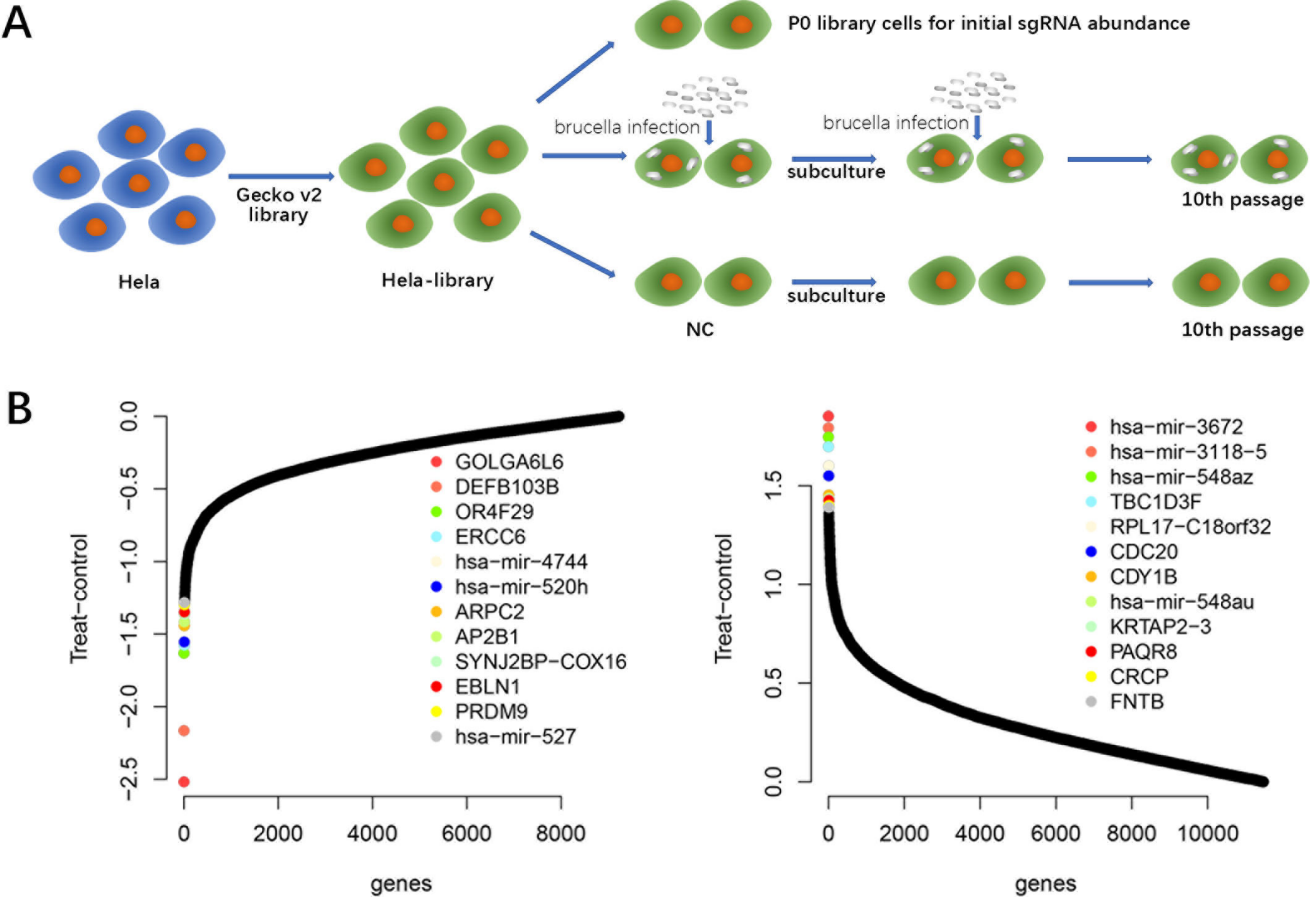
Genome-wide CRISPR knockout screen in *Brucella* infection. (**A**) Schematic representation of the loss-of-function genome-wide screen using the human lentiviral CRISPR/Cas9 library (GeCKOv2) in *Brucella*-infected HeLa cells. (**B**) Top-ranked negative and positive selection genes. *GOLGA6L6* is the first in negative selection, followed by *DEFB103B*.

To identify host genes participating in *Brucella* infection, HeLa cells were transduced with a single guide RNA library containing 19,050 targeted genes in human genome with 123,411 gRNA (34) and cultured for 10 passages with or without *Brucella* infection (Fig. 1A). Prior to CRISPR screening, library cells were tested for *Brucella* tolerability. Pooled knockout cells were infected with *Brucella* at two different multiplicity of infection (MOI) values and counted at 48 hours post infection (P.I.) within each passage. Compared with wild type (WT) HeLa cells, the cell viability of the culture library cells was diminished during *Brucella* infection at both concentrations (Fig. S1A) and suggested the feasibility of CRISPR screening for the identification of key genes in *Brucella* infection. Library cells were nearly halved in viability during MOI 100 infection compared with WT cells but consistently persisted during passaging, whereas complete demise was observed at MOI 1,000 during passaging (Fig. S1A). In accordance with the purpose of the experiment, which was to identify the host gene that impaired *Brucella* infection, regardless of their knockout enhanced or attenuated host cell viability, thus, CRISPR screen of *Brucella* infection required both positive and negative screening results. Consequently, the library cells had to remain viable at all times during *Brucella* infection and enrichment, which prompted the *Brucella* infection to be in conditions of MOI 100.

For CRISPR screen, *Brucella* infection and cell enrichment were performed according to the designated protocol, with the cell number at each passage close to that of the pre-experiment (Fig. 1A; Fig. S1B). At the 3rd,6th, and 10th passage, cells were stained by immunofluorescence for intracellular *Brucella* observation (Fig. S1C).

The relevance of cell viability of the target genes was summarized and generated by sgRNA abundance (Fig. 1B). Surprisingly, most of the detected genes have never been reported relevant to *Brucella* infection. For negative selection, the top-ranked gene *GOLGA6L6* (golgin A6 family like 6) had completely no study for function or mechanism, except for it belonging to the golgin A6 family, localized on human chromosome 15, and highly expressed in testis (35, 36). The top two gene *DEFB103B* (defensin beta 103) functioned as antimicrobial peptide that counteracts Staphylococcus and reduces Mycobacterial infection capacity (37, 38), which was confusing because the purpose of negative selection was infection control, but gene function suggested the presence of *DEFB103B*, not knockout, should have induced bacterial death. The top gene of positive selection (except for microRNA), *TBC1D3F* (TBC1 domain family member 3F), was overexpressed in EOC cell lines and primary tumors compared with normal tissues that could be related to ovarian cancer (39). *Rpl17-C18orf32* represented naturally occurring read-through transcription between the neighboring *RPL17* (ribosomal protein L17) and *C18orf32* (chromosome 18 open reading frame 32) genes and was putatively belonging to the CTCF interactome (40).

### Viability of *GOLGA6L6*-, *DEFB103B*-, *TBC1D3F*-, and *Rpl17-C18orf32*-deficient cells consistent with the CRISPR screen

To verify the results of CRISPR-Cas9 screen, we constructed lentivirus of sgRNAs against *GOLGA6L6*, *DEFB103B*, *TBC1D3F*, and *Rpl17-C18orf32* for gene knockout cell generation. Three sgRNAs of each gene were selected. All cell lines were put through genome DNA sequencing for knockout verification; only the genome mutation sites identical to sgRNA loci were considered successfully knocked out (Fig. S1D). Besides, a clonal of the non-targeting HeLa cell line was generated as a control. The wild-type and non-targeting HeLa cells have identical DNA sequences in the region surrounding the sgRNA target sites of the above genes.

**Fig 2:**
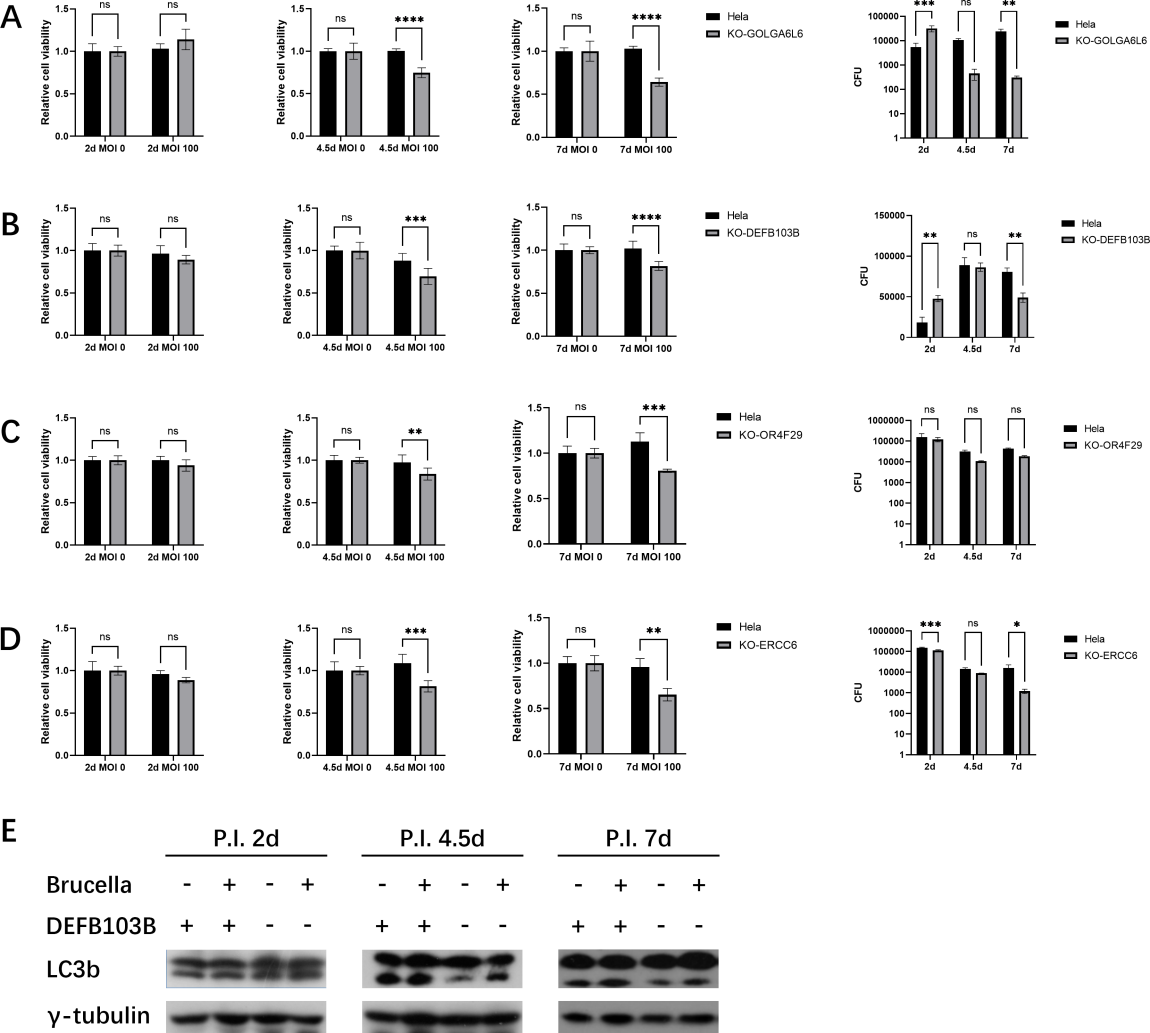
The negative selection genes induced intracellular *Brucella* elimination by declining host cell viability. (**A–D**) The cell viability and intracellular *Brucella* calculation of the negative selected gene knockout cell lines. The four top ranked genes, *GOLGA6L6*, *DEFB103B*, *OR4F29*, and *ERCC6*, were knocked out in HeLa cells with specific sgRNA transfected. A non-targeting sgRNA-transfected HeLa cell was used as negative control. All cells were cultured and infected by *Brucella* under the same conditions of CRISPR screen. Data were represented as mean ± SEM. (**E**) Autophagy detection of the *Brucella*-infected *DEFB103B*-null cells using LC3b. Cells were infected and subcultured with process identical to CRISPR screening and dissociated for western blotting test.

All cell lines were infected by *Brucella* under the same condition in CRISPR screen for 7 days over three passages. Cell viability was tested by CCK8 at each passage. In agreement with CRISPR screening, *GOLGA6L6*- and *DEFB103B*-null cells diminished during subculture under *Brucella* infection compared with HeLa non-targeting cells, while *TBC1D3F*- and *Rpl17-C18orf32*-null cells were amplified (Fig. 2A, B, 3A and B). Uninfected control cells and gene knockout cells were tested for cell viability as blank control.

**Fig 3:**
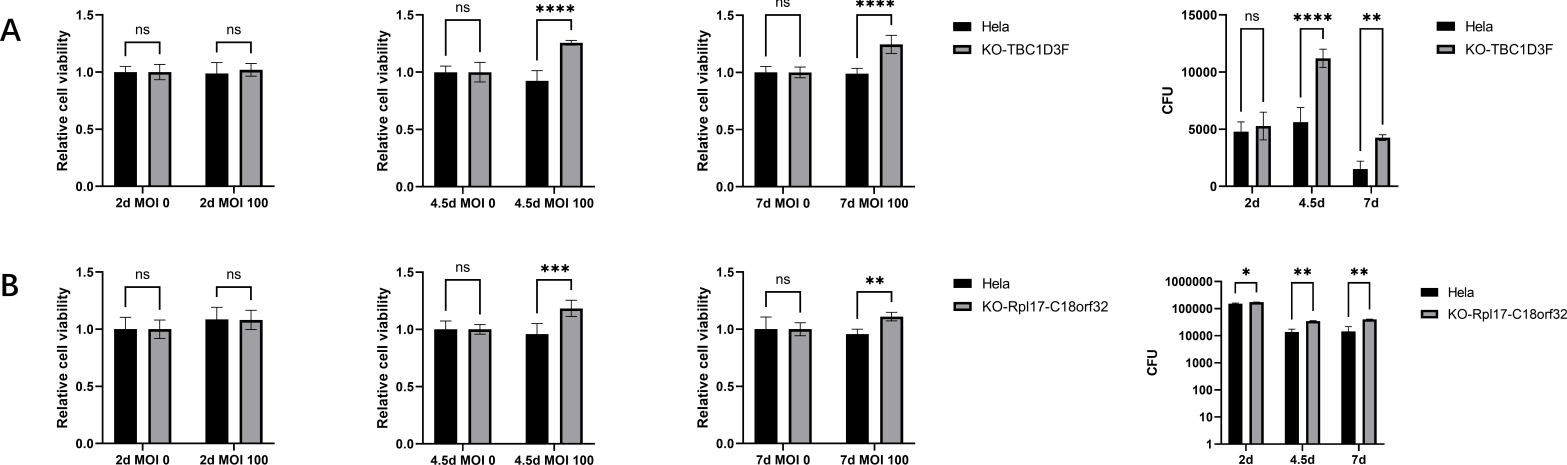
The positive selection genes prolonged host cell lifespan and thus facilitated *Brucella* long-term infection. (**A and B**) The cell viability and intracellular *Brucella* calculation of the positive selected gene knockout cell lines. The top two positive selected genes except miRNA was knocked out for the CRISPR screen validation and showed consistent cell viability. However, the intracellular *Brucella* in these cell lines were also significantly increased, which suggested that knockout of these genes was counterproductive to the control of *Brucella* infection.

### Negative selection gene mutant cells restrained intracellular *Brucella* in long-term infection

The *GOLGA6L6*- and *DEFB103B-*knockout cells were lysed for intracellular *Brucella* culture post cell viability test. CFU calculation suggested intracellular *Brucella* of *GOLGA6L6*- and *DEFB103B*-null cells increased at 2.5 days post infection, but decreased at 4.5 days and 7 days post infection (Fig. 2A and B).

Since knockout of the top-ranked negative selection genes *GOLGA6L6* and *DEFB103B* impacted the intracellular survival of *Brucella*, we further investigated the lower-ranked genes *OR4F29* (olfactory receptor family 4 subfamily F member 29), a gene with olfactory receptor and G-protein-coupled receptor activity, and *ERCC6* (ERCC excision repair 6, chromatin remodeling factor), a gene that encodes a DNA-binding protein important in transcription-coupled excision repair. Results showed *OR4F29*- and *ERCC6-*null cells diminished during *Brucella* infection and subculture, and the intracellular bacteria also decreased (Fig. 2C and D).

### *DEFB103B* affected *Brucella* survival and cell viability by altering cellular autophagy

The functions of most of the genes derived from the screen were not well related to the known mechanisms of *Brucella* infection, and some were even not functionally known, except that *DEFB103B* encoded the defensin beta 103, an antimicrobial peptide with broad-spectrum antimicrobial activity that played an important role in innate epithelial defense. However, in *Brucella* infection, knockout of *DEFB103B* rather facilitated intracellular clearance of *Brucella*, so we hypothesized the host regulation of *DEFB103B* should be conflicted with *Brucella* colonization. Further reviewing suggested *DEFB103B*-attenuated inflammation required autophagy activation (41), which accords with *Brucella* long-term infection (7). Therefore, we monitored host cell autophagy in *Brucella*-infected *DEFB103B* knockout cells. The cellular LC3II were found upregulated in HeLa WT cells upon *Brucella* infection but decreased in *DEFB103B*-knockout cells, which suggested deficiency of *DEFB103B* disrupted a necessary pathway of *Brucella* survival (Fig. 2E). Although LC3II expression was similarly upregulated in *Brucella*-infected *DEFB103B*-knockout cells compared with the uninfected, the level of expression was still lower than that in WT cells (Fig. 2E).

Without *Brucella* infection, there was no significant difference in LC3b between wild-type HeLa cells and *DEFB103B*-null cells at P.I. 2 days, but LC3II decreased at P.I. 4.5 days and 7 days. Since autophagy functioned mostly on promoting cell survival by stabilizing energy metabolism (42), *DEFB103B*-deficient induced inactivation autophagy was possibly the reason of cell viability decline.

### Positive selection gene mutant cells facilitate intracellular *Brucella*

The *TBC1D3F*- and *Rpl17-C18orf32*- null cells were lysed for intracellular *Brucella* culture post cell viability test. CFU calculation suggested intracellular *Brucella* of *TBC1D3F*- and *Rpl17-C18orf32*-null cells increased at 2.5 days, 4.5 days, and 7 days post infection (Fig. 2C and D).

## DISCUSSION

Intracellular infection of *Brucella* is a major cause of brucellosis, threatening public health, and economic property security. Upon infection, *Brucella* may evade host immunity and alter the survival mechanism of host cells to promote its own proliferation. Therefore, revealing the host cellular life activity is the key to controlling *Brucella*. Due to the limitations of the current studies on the host in the *Brucella* infection process, we conducted CRISPR-Cas9 screen at the human genome level to identify genes that directly affect the viability of *Brucella*-infected cells to provide a more comprehensive understanding and to underpin the control of *Brucella* intracellular infection.

CRISPR-based genetic screen was a fine solution of host gene identification related to *Brucella* infection based on cell viability. We tended to characterize genes that promote *Brucella* clearance, which might promote the host cell lifespan by eliminating *Brucella* infection or control *Brucella* infections by excluding infected cells, which corresponded to positive and negative selection of CRISPR screen, respectively. It turned out that while individual knockouts induced cellular phenotypes consistent with genome-wide screening, only knockout of negative selected genes promoted *Brucella* clearance. The positive selected genes prolonged host cell survival along with *Brucella* infection, which even raised the risk of chronic brucellosis.

The *GOLGA6L6*-, *DEFB103B*-, *OR4F29*-, or *ERCC6*-null cells exhibited significant suppression of intracellular *Brucella*, especially in long-term infections, which updated our knowledge about the host genes involved in *Brucella* intracellular survival.

Currently, the function of *GOLGA6L6* remained unknown, but it showed the strongest attenuation of *Brucella* intracellular infection among the screened genes. Since it belonged to the golgin family, we hypothesized it was likely involved in in ER-Golgi trafficking or MHC-I display (24, 43). Intracellular infection contributed to *Brucella* evading of antibiotic clearance. Secretory trafficking of cells provided host ER-derived membrane to the intracellular niche of *Brucella* (24), and in conjunction with studies of ER-related functions and pathways (12, 18, 21), ER-Golgi-associated mechanisms proved critical in intracellular replication of *Brucella*. The infected cells also escaped clearance of host cell immune and in turn established chronic infection, which suggested *Brucella* inhibition of antigen presentation. Barionuevo et al. showed that infection of *Brucella* would not affect the expression of MHC-I in host cells but trapped it within the Golgi to prevent it from presenting to the cell membrane and therefore down-modulated T cell response (43).

*ERCC6* mainly functioned in DNA base excision repair, interstrand cross-link repair, transcription, chromatin remodeling, RNAPII processing, nucleolin regulation, rDNA transcription, redox homeostasis, and mitochondrial function (44), which might be related to *Brucella*-mediated DNA repair (45). It is assumed that intracellular pathogenic bacteria have to cope with DNA alkylating stress within host cells, and the control of DNA repair in *Brucella* displays distinct features (45), which suggested the necessity of DNA repair in *Brucella* infections, but the relevant studies were still scarce. *Brucella* possessed similar DNA repair systems to those of other model bacteria but seemed functionally different (46). The conserved two-component regulatory system BvrR/BvrS in *Brucella* formed an operon with DNA repair genes but has been only found to be involved in metabolic pathways required for intracellular infection (47). Collectively, *Brucella* infection required host DNA repair, but relevant genes in both the host and the bacteria needed to be further investigated. In the CRISPR screen, *ERCC6*, a host DNA repairing gene, was able to affect the intracellular survival of *Brucella*, which suggested that *Brucella* might also achieve DNA repair through host gene regulation.

*OR4F29* was neither functionally studied nor characterized in association with *Brucella*-host interactions, which made it difficult to further parse. Besides, although *OR4F29* knockout cells attenuated *Brucella* infection, the intracellular bacterial count lacked statistical significance. Thus, at the current stage, it mainly diminished host cell viability as well as broadened our knowledge of host genes involved in the regulation of *Brucella* infection.

Of higher relevance to the current studies of *Brucella* would be *DEFB103B*, but it functioned as an antimicrobial peptide in contrast to the results in this study (37, 38). Further investigation revealed that *DEFB103B* execution depended on the activation of autophagy (41), and the decrease in LC3II suggested a significant reduction of cellular autophagy after gene knockout. The current researches generally agreed that autophagy was essential for *Brucella* infection. The use of multiple autophagy inhibitors, such as 3-MA or chloroquine, also suggests that autophagy was essential for *Brucella* colonization (48, 49). The BCV acquired autophagic features at late stages of the intracellular life cycle and promoted cell-to-cell spreading of *Brucella* (7). The requirement of autophagy-initiation proteins ULK1, Beclin 1, and ATG14L and PI3-kinase activity but not elongation proteins ATG5, ATG16L1, ATG4B, ATG7, and LC3B suggested *Brucella* selectively co-opts autophagy-initiation complexes to subvert host clearance and promote infection (5, 7). The studies of Verbeke et al. revealed that not only conventional autophagy but also mitophagy was indispensable for *Brucella* egress among host cells (50). The functionality of the multiple type four secretion system effector proteins and the LysR-type transcriptional regulators has also been found to be associated with autophagy that ultimately affects the intracellular survival of *Brucella* (49, 51, 52). Altogether, *DEFB103B* deletion-mediated host cell autophagy attenuation was most likely responsible for the decline of *Brucella* intracellular survival. In addition, the *DEFB103B* knockout cell line showed an upregulation of autophagy levels after infection, which suggested that *Brucella* regulated cell autophagy through multiple pathways.

According to the study of Peng et al., *DEFB103B* alleviated the IL-4- and IL-13-mediated impairment of the tight junction (TJ) barrier through keratinocyte autophagy activation, which involved aryl hydrocarbon receptor (AhR) signaling (41), whereas the studies of Wong et al. suggested that autophagy, as a cell survival mechanism, enhances intestinal epithelial TJ barrier function (53, 54). Therefore, the decline of cell viability in *DEFB103B*-null cells was probably induced by the inactivation of autophagy, which rendered *DEFB103B* as a potential brucellosis therapeutic target. Interference of *DEFB103B* could attenuate host cell survival and thus control *Brucella* intracellular infection.

Although genome-wide CRISPR screens have improved our understanding of host regulation following *Brucella* infection, the technique could only be used to screen genes that had an impact on cell viability. This phenotype, although beneficial in the control of *Brucella* infection, still had limitations in the resolving gene regulation of the host post infection. During the screening, genes involved in the regulation of *Brucella* infection but not in modifying cell viability will be eliminated; hence, the host-*Brucella* interaction still required further exploration.

In conclusion, we performed a genome-wide CRISPR Cas9 screen of *Brucella*-infected cells and firstly identified *GOLGA6L6*, *DEFB103B*, *OR4F29*, and *ERCC6* as potential targets for intracellular *Brucella* control. Among which, *DEFB103B* is an antibacterial peptide that activates autophagy, and its knockout significantly reduced host autophagy as well as intracellular bacterial survival.

## MATERIALS AND METHODS

### Bacteria and cell lines

HeLa and HEK293T cells were cultured in Dulbecco’s modified Eagle’s medium (DMEM from EallBio, 03.1006C) with 25 mM glucose, 10% fetal bovine serum (FBS from Every Green, 11011–8611), and 100 U/mL penicillin/streptomycin under 5% CO_2_ at 37°C. *Brucella melitensis 16M* was laboratory conserved.

### *Brucella* infection

Firstly, a freshly streaked single *Brucella* colony was inoculated in 5 mL of TSB and cultured at 37°C. The optical density for the bacterial solution at 600 nm was measured for *Brucella* calculation, and infections were performed at a multiplicity of infection of 100:1. Cells were washed three times with PBS and changed into DMEM without antibiotics during 2 hours of infection and then cultured in DMEM with 10% FBS and gentamicin (10 mg/mL) for extracellular bacterial elimination.

### Genome-wide CRISPR screen for *Brucella* infection

The human GeCKOv2 CRISPR knockout pooled library used in this study was a gift from Feng Zhang (Addgene#). Construction and lentivirus production were performed as previously described (33, 34). HeLa cells were seeded in two T175 flasks and infected by lentivirus for genome-wide knockout library at MOI 0.3. Puromycin (2 µg/mL) selection began 24 hours post lentiviral transduction. The *Brucella* infection screen was set up following 7 days of puromycin selection, at which point 2.4 × 10^7^ HeLa-library cells were collected for the analysis of the initial sgRNA abundance. Then, 4.8 × 10^7^ HeLa-library cells were seeded in 20 T175 flasks; 10 of which were infected by *Brucella*, and the rest were cultured as negative control (NC). Two days post infection, HeLa-library cells were digested, collected, and reseeded in T175 flasks for subculture at the same density (respectively, *Brucella* infected and NC). Both groups of cells attained 10 serial passages for phenotype enrichment and then were harvested for DNA extraction and sgRNA analyzing.

### Immunofluorescence detection

Cells were fixed with 4% paraformaldehyde for 20 min, incubated with 0.1% Triton X-100 for 5 min, and then blocked with 5% BSA for 1 hour at room temperature. Next, cells were incubated with *Brucella* antibody (Bioss, bs-2229R) and FITC-conjugated secondary antibody (Invitrogen, F-2765) according to the manufacturer’s protocol. Then, phalloidin (Invitrogen, A12381) and DAPI (Bestvio, BB-4401) were stained respectively for F-actin and the nucleus.

### Generation of knockout cell lines

sgRNA oligonucleotide pairs against the target genes (Table 1) were synthesized, annealed, and cloned into the BsmBI-linearized lentiCRISPRv2 plasmid. As a control, a non-targeting sgRNA was also cloned. These sgRNA-Cas9-containing vectors were transiently transfected with pSPAS2 and pMD2G by polyetherimide into HEK293T cells for lentivirus generation and then HeLa cell infection. After 48 hours of puromycin selection, cells were collected for preservation and knockout identification.

**TABLE 1:** sgRNA sequences for gene knockout and primers for knockout identification

Gene	sgRNA	Fp sequence	Rp sequence	Product size
*GOLGA6L6*	TACCAACACTAGGGTTGGTC	CAGTTGGTGGCATTAAATCAGA	CAGAAGTGGTTGTCTCAGGGTT	222
	GAAGGCAAGCCATCAACATC	ATGTCTCTTGCTGTGCCATCT	ATTCGTATGGTATGAACCTGGG	225
	GACTCACATCCTCAGGCGAG	TTTCCACAGTTGACAGACCATC	TGGCAGTTCTTCCACAATCTTA	223
*DEFB103B*	ATCGGCAAGTGCTCGACGCG	GCAAGGGATGAGTTATTTGAGG	ACTCTCGTCATGTTTCAGGGTT	264
	TTGCCGATCTGTTCCTCCTT	GCAAGGGATGAGTTATTTGAGG	ACTCTCGTCATGTTTCAGGGTT	264
	CTGGAACAGGCACCAAAAAC	CACACCTTTTCATCCAGTCTCA	CCTGGCCTCTTAATGATTCTTG	264
*TBC1D3F*	AGGGCCCTCTACGTCCAGGT	CAGGGTTCCTGGAGATTCCT	CTGTTCGTCCCTAGCTCTGAAG	247
	TTGGTGGGCACAAGAGCGAG	TGGGACTTGAGACTCCAGAGA	AAGAAGCAGACCGACTTGTACC	279
	TTCGCCTCCCGCGCAGTCAG	GTGCAGTTCCTCAGCTCTGC	TGTGGTTCTCCTTTTGGAATTT	279
*Rpl17-C18orf32*	GCCATATACGACTAACGAAG	TGCATTCCTTGTATCGTCATTC	AGTGTTTCACCCAAGTACCACC	222
	TTGTGTCCAGCCCCATTGCT	GCTGTAATGACAGGACACCTTG	TTTAAGCATGTGCAGCAAAAAT	209
	CAACTCCACCATTGTAACGT	TATGCATATACGAAAAGCCACG	GTCCTGTCATTACAGCAACCAG	222
*OR4F29*	GATAGTGGAGGGGCTTACAT	ACTTAGGAGCCTGCTCTGTCAC	TTAACAAGAAATGCCAGTTGGA	286
	CAGTCACTCCCTGTTCCAAC	TTGACAGATATGTGGCCCTATG	ACAGTGACCATGAACTGCAATC	244
	CTTGTTAATTTAGCCTTCTG	TTGACAGATATGTGGCCCTATG	ACAGTGACCATGAACTGCAATC	244
*ERCC6*	ATGTCGGTCGATGTGCAGCA	GGTGGAGGAGTACCTCTCCTTT	CGATACTCCTTCTCCACGTCA	282
	TGAACTAGATCACGCCAGTC	TCACTGCAAAACAAAAGCATCT	TTGGTTCTGCAGGTGTAGAGAA	260
	GGTCATGTACGACATCCCTA	TTTTATTTGTGGCCAATAAGGG	TTTCCTGTTGATGTCTCTGCTG	247
Non-targeting	GTATTACTGATATTGGTGGG			

The genomic DNA of HeLa WT, non-targeting knockout HeLa cells, and the gene knockout cell lines was extracted by the Mouse Direct PCR Kit (Bimake, B40013) according to the manufacturer’s protocol; only the mouse tail was replaced by 1 × 10^6^ cells. A DNA region surrounding the sgRNA of the target genes was PCR amplified using primers listed in Table 1. The PCR product was purified and cloned using 5 min Universal Ligation Mix (Vazyme, C311-01). *Trelief* 5α competent cells (Tsingke, TSC-C01) were transformed with the ligation mixture and cultured on LB ampicillin agar plates. At least 15 colonies for each sgRNA were picked for ligation inspection by PCR. The DNA was then sequenced to identify whether a Cas9-mediated loss-of-function mutation was generated on both alleles in the potential knockout clones.

### Cell viability assay

Cell viability was monitored with the CCK8 Kit (CCK8, Dojindo, Japan) with slight modification of the producers’ protocol. Cells were previously seeded in six-well plates and conducted *Brucella* infection. Two days post infection, cells of each single well were digested and collected in 1.5 mL of culturing medium. Then, 30 µL of collected cells, 10 µL of CCK8 reagent, and 60 µL of cell culturing medium were mixed well and added to a well of a 96-well plate to serve as a CCK8 testing well. Six parallels were prepared for each tested cell line. The rest procedures were identical to the manufacturers’ instructions. Absorbance at 450 nm was measured by a microplate reader (Thermo Scientific, Multiskan FC).

### Intracellular bacterial culture

The rest cells after CCK8 were centrifuged at 300 × *g* for 5 min and washed with PBS three times. Then, cell pellet was resuspended with 0.1% Triton X-100 in water and incubated at room temperature for 5 min. The lysate was spread on a TSA plate and placed at 37°C for bacterial culture. The CFU of each plate was counted for intracellular bacterial calculation.

### Western blotting

Cells lysates were generated using 5× SDS-PAGE sample buffer (Solarbio, P1043). The samples were boiled at 100°C for 10 min and then loaded with 20 µg of BCA-quantified protein (Thermo Scientific, A55864). Proteins were resolved on SDS-PAGE and transferred onto 0.45 µm nitrocellulose membranes (Cytiva, 10600003). Membranes were blocked in 5% (wt/vol) non-fat dry skim milk powder in TBST for 1 hour at room temperature and probed with primary antibodies overnight at 4°C and then with HRP-conjugated secondary antibodies. All antibodies were diluted in blocking buffer according to the manufacturers’ protocol. The images were obtained by scanning the film after exposure in a darkroom, and representative figures were assembled using Adobe Photoshop 2021.

### Statistical analysis

Data are presented as the mean ± SEM. Samples were not excluded from the analysis, randomization did not occur, and investigators were not blinded. Statistical significance was determined using one-way analysis of variance (ANOVA) with a Bonferroni post test correction and two-way ANOVA when two variables were present. Statistical significance was determined at the 0.05 level.

### Oligo sequences

Sequences of sgRNAs for the knockout of the targeted genes were listed in Table 1. Primers for amplification of genomic DNA flanking sgRNA were also listed.

## Data Availability

The authors declare that all data supporting the findings of this study are available within the paper and the supplementary figure. No data sets were generated or analyzed during this study.

## SUPPLEMENTAL MATERIAL

Fig. S1 spectrum.03383-23-s0001.tif Quality control of screening process and the sequencing of knockout genes.

ASM does not own the copyrights to Supplemental Material that may be linked to, or accessed through, an article. The authors have granted ASM a non-exclusive, world-wide license to publish the Supplemental Material files. Please contact the corresponding author directly for reuse.
