# Supplementary figures and images for: A genome-wide CRISPR screen identified host genes essential for intracellular Brucella survival
Source: Microbiol Spectr. 2024 Feb 20;12(4):e03383-23. doi: 10.1128/spectrum.03383-23 (PMC10986529; doi:10.1128/spectrum.03383-23)

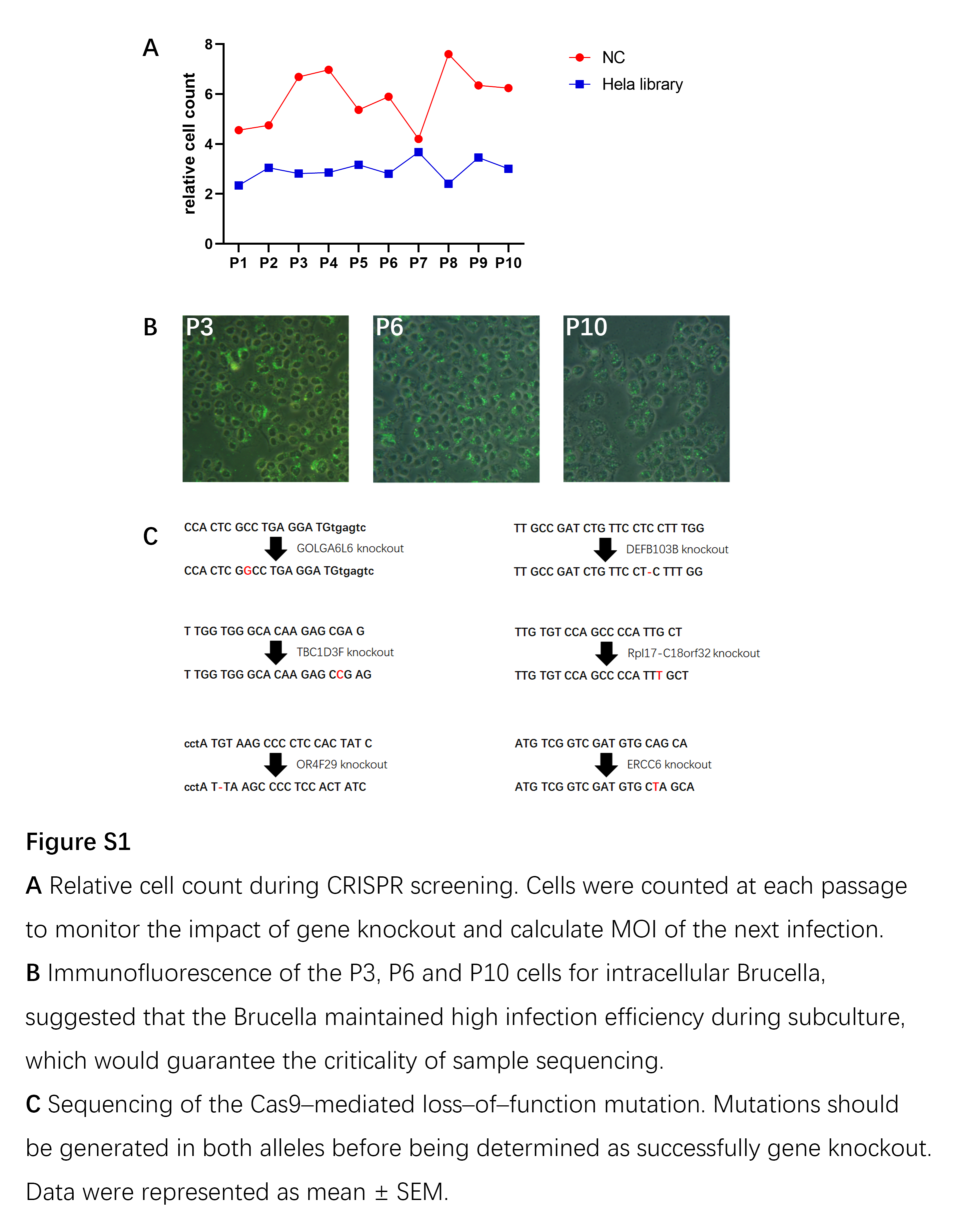

Supplement: Fig. S1 — Quality control of screening process and the sequencing of knockout genes. [file spectrum.03383-23-s0001.tif]
